# Small RNAs in eucaryotes: new clues for amplifying microRNA benefits

**DOI:** 10.1186/s13578-019-0370-3

**Published:** 2020-01-03

**Authors:** Bernardetta Ledda, Laura Ottaggio, Alberto Izzotti, Samir G. Sukkar, Mariangela Miele

**Affiliations:** 1 Department of Health Sciences, University of Genoa, Via A. Pastore 1, 16132 Genoa, Italy; 2 Mutagenesis and Cancer Prevention Unit, IRCCS Ospedale Policlinico San Martino, L.Go R. Benzi, 10, Genoa, Italy; 3 UOD Dietetic and Clinical Nutrition, IRCCS Ospedale Policlinico San Martino, L.Go R. Benzi, 10, Genoa, Italy

**Keywords:** microRNA, Small non coding RNA, Plant miRNA, Food miRNA, miRNA biogenesis

## Abstract

miRNAs, the smallest nucleotide molecules able to regulate gene expression at post transcriptional level, are found in both animals and plants being involved in fundamental processes for growth and development of living organisms. The number of miRNAs has been hypothesized to increase when some organisms specialized the process of mastication and grinding of food. Further to the vertical transmission, miRNAs can undergo horizontal transmission among different species, in particular between plants and animals. In the last years, an increasing number of studies reported that miRNA passage occurs through feeding, and that in animals, plant miRNAs can survive the gastro intestinal digestion and transferred by blood into host cells, where they can exert their functions modulating gene expression. The present review reports studies on miRNAs during evolution, with particular focus on biogenesis and mechanisms regulating their stability in plants and animals. The different biogenesis and post biogenesis modifications allow to discriminate miRNAs of plant origin from those of animal origin, and make it possible to better clarify the controversial question on whether a possible cross-kingdom miRNA transfer through food does exist. The majority of human medicines and supplements derive from plants and a regular consumption of plant food is suggested for their beneficial effects in the prevention of metabolic diseases, cancers, and dietary related disorders. So far, these beneficial effects have been generally attributed to the content of secondary metabolites, whereas mechanisms regarding other components remain unclear. Therefore, in light of the above reported studies miRNAs could result another component for the medical properties of plants. miRNAs have been mainly studied in mammals characterizing their sequences and molecular targets as available in public databases. The herein presented studies provide evidences that miRNA situation is much more complex than the static situation reported in databases. Indeed, miRNAs may have redundant activities, variable sequences, different methods of biogenesis, and may be differently influenced by external and environmental factors. In-depth knowledge of mechanisms of synthesis, regulation and transfer of plant miRNAs to other species can open new frontiers in the therapy of many human diseases, including cancer.

## Background

Non-coding RNAs (ncRNAs) are RNAs that do not encode proteins; however, such RNAs contain biological information controlling various levels of gene expression, including chromatin architecture, epigenetics, transcription, splicing, editing, and translation [1–3]. More and more evidences indicate that the majority of mammalian and other complex organism genomes are transcribed into ncRNAs.

ncRNAs include long ncRNAs formed by more than 200 nt, small ncRNAs (sncRNAs) formed by less than 200 nt, and a variety of other transcripts most of which with unknown function [4]. Among sncRNAs it is possible to distinguish microRNAs (miRNAs) and short interfering RNAs (siRNAs) [5], miRNAs and siRNAs are single-stranded molecules about 18–22 nt and 20–24 nt long, respectively, both deriving from cutting of long dsRNA stem loop precursors. However, small RNAs other than miRNAs have been identified and characterized as not deriving from stem loop precursors [6–8]. sncRNAs act directing specifically the binding of effector proteins on the target nucleic acid through base pairing interaction. miRNAs and siRNAs with function of endogenous gene regulation and genome protection from exogenous nucleic acid attack, respectively, are widely present in eukaryotic cells [9]. miRNAs and siRNAs are very similar to each other and it was thought that the main difference between them was in the origin: miRNAs being endogenous and siRNAs being exogenous; however, it is currently known that there are also many endogenous siRNAs. Indeed, the main differences between them are the precursor structure, the pathway of biogenesis and the mechanism of action [10]. Both miRNAs and siRNAs bind to complementary sites in their target mRNA, regulating post-transcriptionally gene expression in both plants and animals. Typically, miRNAs negatively regulate targets by mRNA cleavage in trans, inhibiting translation. Plants also use miRNAs and siRNAs to regulate target genes in response to abiotic and biotic stress [11]. sncRNAs have been found in different species, both in animals and in plants, and several studies reported that they are strongly preserved from plants to metazoa.

The present review concerns the role of sncRNAs during evolution, with particular focus on miRNA biogenesis and mechanisms regulating their stability in plants and animals. The different biogenesis and post biogenesis modification is one of the ways to discriminate miRNAs of plant origin from those of animal origin, and make it possible to better clarify the studies on the possible flow of miRNAs from plants to animals. The hypothesis on whether a possible cross-kingdom miRNA transfer through food does exist, and on whether human gene expression could be influenced by dietary uptake of plant miRNAs gave rise to a controversial question, today still unresolved. Then, the deepening aimed at shedding light on the molecular mechanisms performed in different species could improve sncRNA translatability in humans from bench to bedside for their potential application prospects in nutrition and medicine, making it possible the therapeutic exploitation of these molecules in treating human diseases.

## sncRNAs in animals and plants

Only one-fifth of transcription across the human genome is associated with protein-coding genes by producing protein coding RNAs. However, it is increasingly accepted that other small RNAs, not directly involved in these functions, do exist. Among sncRNAs, miRNAs and siRNAs play a fundamental role as master regulators of gene transcription at post-transcriptional level. They are the transcription products of genes whose number changes in different species, and guide a complex of proteins to complementary mRNA targets, a process resulting in mRNA disruption. The processing and effector silencing proteins for miRNAs and siRNAs are proteins belonging to Argonaute (AGO) and Dicer (DCR) family [12–14]. The biogenesis and activity of miRNAs are strongly related to those of siRNAs that mediate RNA interference, the latter being an ancestral mechanism selected to achieve gene silencing [15]. siRNAs were firstly described in plants since 1990 [16] and it is thought they are much more effective than in mammals due to longer evolution time and to the need of plants to fight invasion from foreign organisms in the absence of a developed immune system [17, 18].

In both animals and plants miRNAs and siRNAs regulate gene expression cleaving mRNA or repressing translation. siRNAs can guide nuclease complexes to cognate mRNAs, which they cleave. siRNAs are derived from either mRNAs of protein-coding genes or long ncRNAs. miRNAs mainly operate through two different mechanisms: miRNAs with high complementarity use cleavage mechanism, while miRNAs with partial or minor complementarity use translational repression [19]. Furthermore, AGO proteins can form RNA-Induced Silencing Complex (RISC) with miRNA precursor single stranded RNA (pre-miRNA), DNA and long unstructured single-stranded RNA [20, 21]. Both animals and plants use a combination of these two methods, alternating cleavage and translational repression, without any cell or tissue specificity, although the former mechanism is mainly adopted in plants and the latter is preferentially adopted in animals.

The majority of plant miRNAs interact with the internal regions of target mRNAs through perfect or near perfect base-pairing to cleave mRNAs; however, some exceptions do exist. For example, miR172, although having a perfect or near-perfect target mRNA sequence complementarity, inhibits the expression of target genes by binding to a unique site [22]. Some miRNAs are preferentially expressed in specific tissues and regulated by developmental switching, including those produced in tissues in response to phyto-hormons and other environmental-stress related elicitors [23]. Biotic or abiotic stressors may function as signals to control and regulate miRNA genes, thus inducing stress-response signals, which in turn phosphorylate transcriptional factors, triggering down or over-expressing the targeted miRNA genes [24]. miRNAs are more strongly expressed in flowers and leaves suggesting the importance of their role in plant growth [22]. Differences and similarity between plant and animal miRNAs have been underlined by several authors, and recently reviewed [25, 26]. Table 1 highlights the main different features of plant and animal miRNAs.

**Table 1:** Plant and animal miRNAs

Characteristics	Plant miRNAs	Animal miRNA
Complementarity	Almost perfect base-pairing	Usually non-perfect
Gene Targeting	Coding region in the open reading frame	3′ untranslated region
mRNA targeting	Single	Multiple
Major mechanisms of action	Cleavage of mRNA target or inhibition of transcription	Inhibition of translation
Grouping	Often belong to large miRNA gene families	Association in large families is uncommon
Length	Short	Long
RNA Polymerases involved	RNA Pol II e III	RNA Pol II
Preservation during evolution	Often conserved among species	Conservation between species is less common than plants

## miRNA biogenesis

**Fig. 1:**
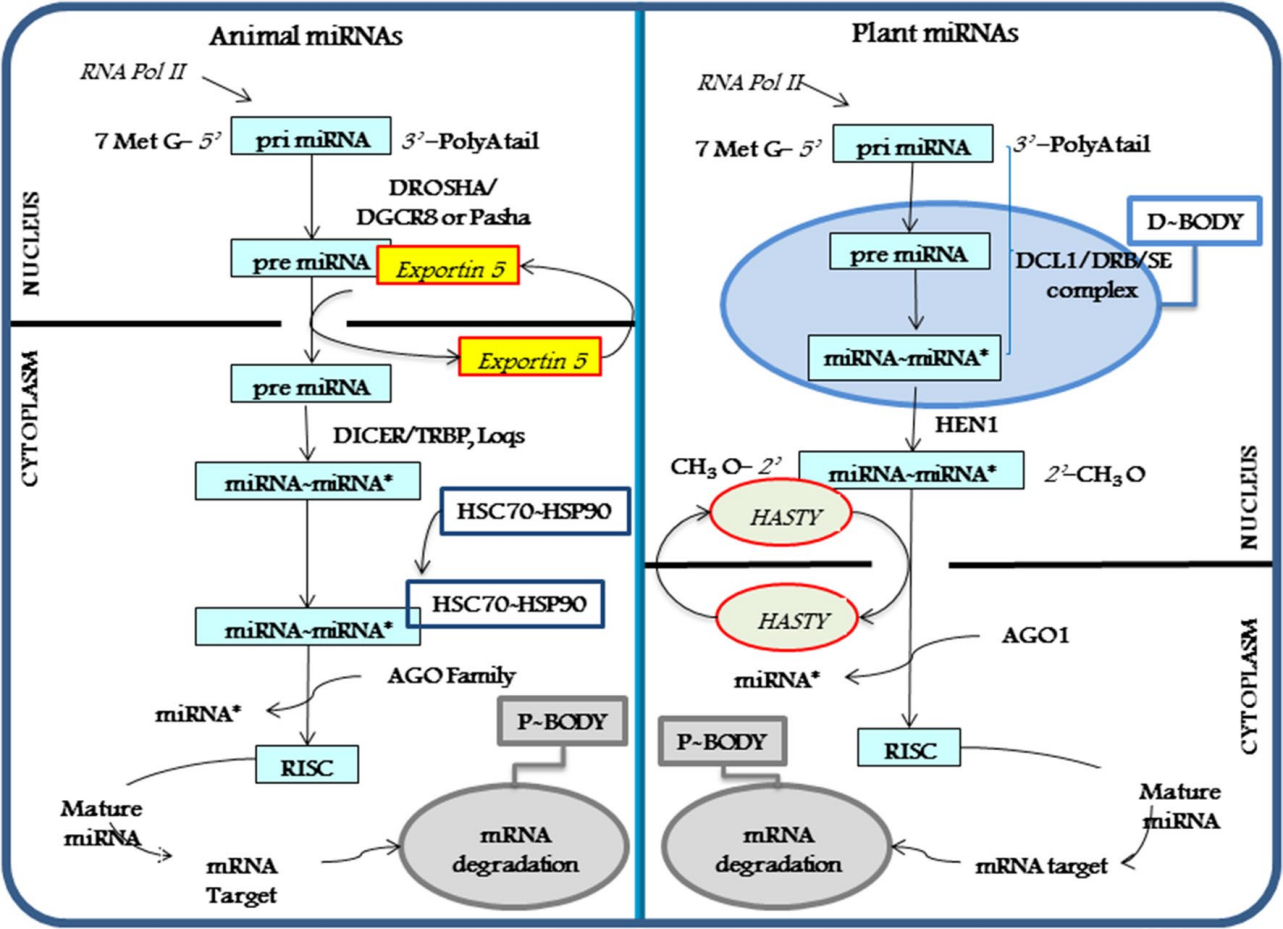
Canonical and non-canonical miRNA biogenesis pathway in animals (left) and plants (right)

In the last years many progresses have been made to understand miRNA biosynthesis pathway in both animals and plants. Accordingly, a canonical and a non-canonical miRNA biogenesis mechanism have been proposed [27–29]. Canonical biogenesis leads to primary miRNAs (pri-miRNAs), transcribed by RNA polymerase II [30], a miRNA subset is also produced by RNA polymerase III [31]. In animals pri-miRNAs capped by a 7-methylguanosine at 5′ end, and polyadenilated at 3′ end, contain a stem-loop structure that is cleaved by Drosha RNase III, in the nucleus. The resulting pre-miRNAs are exported from nucleus to cytoplasm by Exportin 5, then cleaved by DCR enzyme operating in combination with dsRNA-binding partners. This process results in the production of miRNA–miRNA* duplexes, miRNA* indicating the strand that will be eliminated (Fig. 1, left panel). Subsequent maturation steps expel miRNA*, producing a mature RISC, an effector complex targeting and silencing mRNA transcripts. Drosha enzyme is absent in plants, where its function is carried out by the plant DCR-like (DCL) 1 RNase-III, located in the nucleus. DCL1 catalyses both pri-miRNA to pre-miRNA and pre-miRNA to miRNA:miRNA* duplex processes, occurring in specialized sub-nuclear regions termed D-bodies [32, 33] (Fig. 1, right panel). This mechanism is quite different from that adopted by animals, which complete miRNA/miRNA* biogenesis in the cytoplasm. The 3′ nucleotides of the initial miRNA/miRNA* duplex are 2′-*O*-methylated by the nuclear Hua Enhancer-1 protein [34]. This modification prevents non-templated 3′-polymerization that accelerates miRNA turnover [35]. HASTY, the plant homolog of Exportin 5, exports miRNA/miRNA* duplexes to cytoplasm for loading onto AGO proteins [32], acting as a ‘slicer’ to direct the endonucleolytic cleavage of target mRNAs [36, 37]. In both animals and plants there are several alternative miRNA biogenesis, referred as non-canonical, giving rise to a subset of miRNAs having different origins. In animals, the major non-canonical mechanism is represented by mirtrons [38, 39]. On the contrary, plants can process long inverted repeat transcripts into small RNAs, and plant hairpins are generally processed by DCL proteins [40, 41]. Binding of AGO-miRNA complex to mRNA targets regulates gene expression, but also alters the stability of miRNA itself [42, 43]. Binding to RNA targets can stabilize miRNAs by recruiting miRNA protective factors or translocating miRNAs to a subcellular location that lacks small RNA-degrading nucleases. Alternatively, targeted RNAs may protect miRNAs from a release factor that would expel them from RISC and expose them to subsequent destruction [44, 45]. Some authors suggested that in vivo many expressed miRNAs reside in an inactive reserve, which allow resting cells to employ miRNAs to regulate translation in their environment [46]. In plants, high complementarity of miRNAs with their targets triggers miRNA cleavage; however, at least some miRNAs can block translation. By contrast, just a few miRNA–target pairs in mammals have sufficient complementarity to direct AGO protein to cleave the target. miRNA-mediated control in animals occurs in a combinatorial way, with a number of multiple binding sites in mRNA, reflecting the degree of potential repression [25, 47, 48].

Plants, lacking a specific immunosystem, adopted miRNAs to defend against invasion from foreign genomes of infective and weed agents [49].

## miRNAs and evolution

miRNA machinery was pivotal in all evolutionary steps of eukaryotes since the appearance of the first common ancestor of both plants and animals in very early evolution [50–52]. Several miRNAs differ each other for only one nucleotide; this allowed to divide them into families whose number is in continuous progress. miRNAs are evolutionary ancient small RNAs developed in plants 400 million years ago, and only subsequently in animals [53–55]. The appearance of miRNAs has been essential for the evolution of complex organisms. New gene sequencing techniques allowed identifying the miRNA genes conserved during the evolution among the different species. The use of these techniques makes it possible to establish the existence of a direct correlation between the number of miRNAs and the morphological complexity of organisms. This statement arises from the hypothesis that the number of miRNAs has started to increase when some organisms specialized the process of mastication and grinding of food, thus starting a precise process of morphological evolution, and during early bilateral evolution [56, 57].

miRBase (http://www.mirbase.org), the reference miRNA data base, catalogues miRNAs as single sequence only; however, sequence data of small RNAs in various organisms, tissues and cell types show that miRNAs comprise multiple isoforms, also known as isomirs [58, 59]. Such sequence heterogeneity may arise from imprecise precursor cropping or dicing, terminal trimming or the addition of non-templated nucleotides. The 5′ end of a miRNA defines its seed sequence and a single nucleotide shift at this site radically alter its target repertoire.

**Fig. 2:**
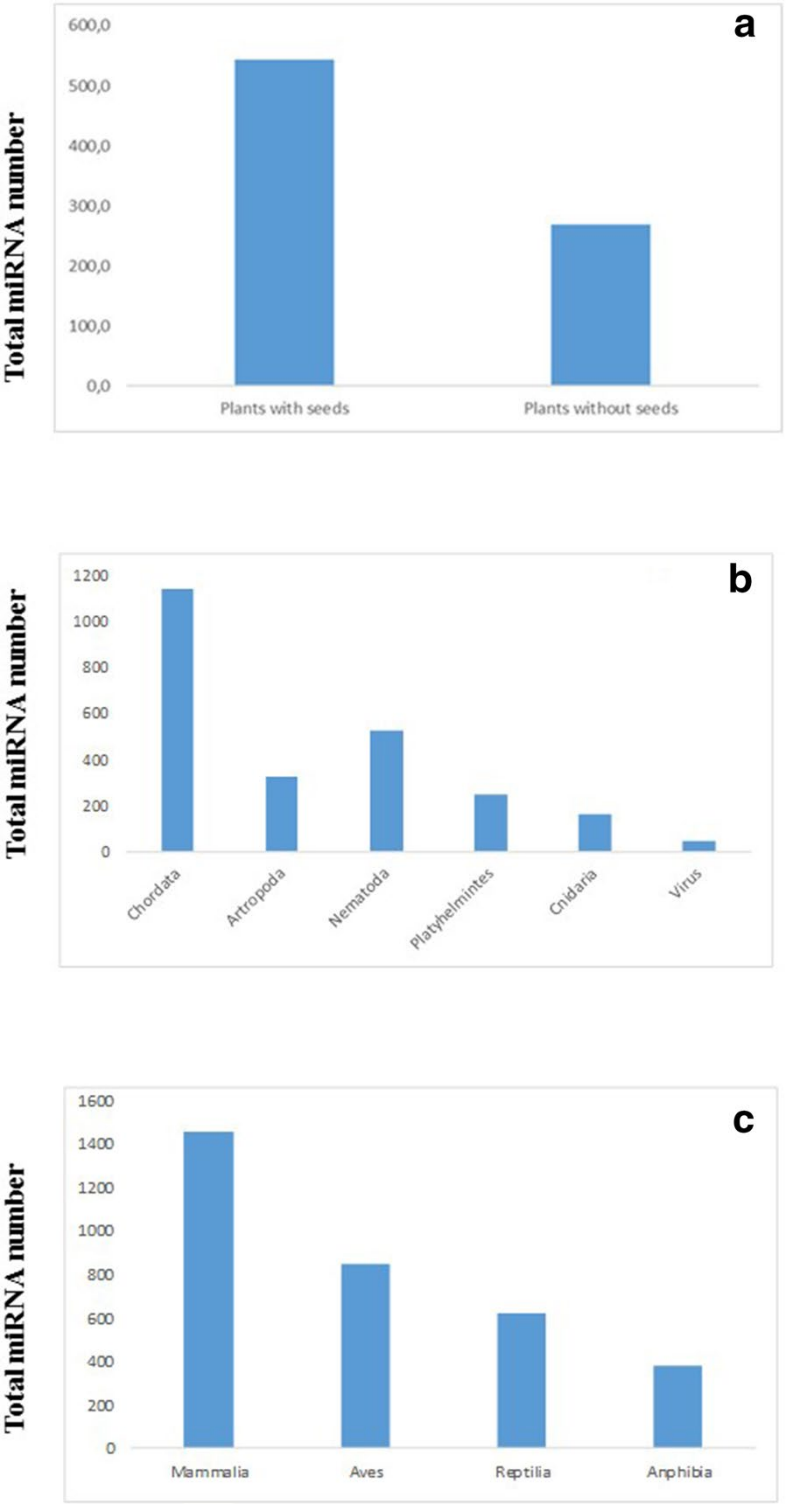
miRNA in plants and animals. Comparison of miRNA number in: plants with seeds and without seeds (**a**); different animal phyla and viruses (**b**); and different Chordata classes (**c**)

Tables 2, 3 and 4 show miRNA number of organisms belonging to different groups. The hairpin precursors and the mature miRNA number in plants, different animal phyla and viruses are reported, as provided by the public miRBase. Table 2 reports data related to different divisions of plant kingdom. The highest levels of miRNAs are found for Magnoliophyta and Pinophyta with an average miRNA number of about 543. These two divisions belong to Spermatophyta superdivision, a group including plants developing through seeds, known to be more evolved than other divisions that have an average miRNA number of about 269 (Fig. 2a).

**Table 2:** Hairpin precursor and mature miRNA number in different plant divisions

ID	Superdivision	Division	Class	Species	Hairpin precursor	Mature miRNA
*mtr*	Spermatophyta	Magnoliophyta	Magnoliopsida	*Medicago truncatula*	710	790
*gma*		Magnoliophyta	Magnoliopsida	*Glicine max*	685	756
*osa*		Magnoliophyta	Liliopsida	*Oryza sativa* (Asian rice)	684	757
*bdi*		Magnoliophyta	Liliopsida	*Brachypodium distachyon*	328	536
*ptc*		Magnoliophyta	Magnoliopsida	*Populus trichocarpa*	364	401
*ath*		Magnoliophyta	Magnoliopsida	*Arabidopsis thaliana*	329	430
*lja*		Magnoliophyta	Magnoliopsida	*Lotus japonicus*	299	365
*gra*		Magnoliophyta	Magnoliopsida	*Gossypium raimondii*	296	296
*aly*		Magnoliophyta	Magnoliopsida	*Arabidopsis lyrata*	206	385
*stu*		Magnoliophyta	Magnoliopsida	*Solanum tuberosum*	224	343
*zma*		Magnoliophyta	Liliopsida	*Zea mays*	174	325
*sbi*		Magnoliophyta	Liliopsida	*Sorghum bicolor*	211	241
*nta*		Magnoliophyta	Magnoliopsida	*Nicotiana tabacum*	207	224
*vvi*		Magnoliophyta	Magnoliopsida	*Vitis vinifera*	168	186
*sly*		Magnoliophyta	Magnoliopsida	*Solanum lycopersicum*	112	147
*atr*		Magnoliophyta	Magnoliopsida	*Amborella trichopoda*	124	129
*cpa*		Magnoliophyta	Magnoliopsida	*Carica papaya*	79	81
*rco*		Magnoliophyta	Magnoliopsida	*Ricinus communis*	63	63
*rgl*		Magnoliophyta	Magnoliopsida	*Rehmannia glutinosa*	32	37
*sof*		Magnoliophyta	Liliopsida	*Saccharum officinarum*	16	16
*aau*		Magnoliophyta	Magnoliopsida	*Acacia auriculiformis*	7	7
*ama*		Magnoliophyta	Magnoliopsida	*Acacia mangium*	2	3
*pab*		Pinophyta	Pinopsida	*Picea abies*	594	600
*cln*		Pinophyta	Pinopsida	*Cunninghamia lanceolata*	5	4
*ppt*		Bryophyta	Bryopsida	*Physcomitrella patens*	250	298
*cre*		Chlorophyta	Chlorophyceae	*Chlamydomonas reinhardtii*	51	86
*smo*		Lycopodiophyta	Selaginellopsida	*Selaginella moellendorfii*	58	64

**Table 3:** Hairpin precursor and mature miRNA number in different animal phyla

ID	Phylum	Class	Species	Hairpin precursor	Mature miRNA
*hsa*	Chordata	Mammalia	*Homo sapiens*	1984	2693
*mmu*		Mammalia	*Mus musculus*	1303	2013
*gga*		Aves	*Gallus gallus*	907	1238
*bta*		Mammalia	*Bos taurus*	1085	1045
*mdo*		Mammalia	*Monodelphis domestica*	681	1139
*ptr*		Mammalia	*Pan troglodytes*	685	690
*ppy*		Mammalia	Pongo pygmaeus	655	673
*rno*		Mammalia	*Rattus norvegicus*	501	769
*oan*		Mammalia	*Ornithorhyncus anatinus*	396	640
*cfa*		Mammalia	*Canis familiaris*	504	455
*ocu*		Mammalia	*Oryctolayus cuniculus*	306	579
*ssu*		Mammalia	*Sus scrofa*	414	461
*cgr*		Mammalia	*Cricetus griseus*	245	353
*sha*		Mammalia	*Sarchophilus harrisii*	67	65
*meu*		Mammalia	*Macropus eugenii*	3	3
*gga*		Aves	*Gallus gallus*	907	1238
*cli*		Aves	*Columba livia*	248	420
*tgu*		Aves	*Taenopygia guttata*	247	334
*apl*		Aves	*Anas platyrhynchos*	4	8
*aca*		Reptilia	*Anolis carolinensis*	303	449
*cpi*		Reptilia	*Chrysemys picta*	268	405
*ami*		Reptilia	*Alligator mississipinensis*	242	373
*oha*		Reptilia	*Ophiophagus hannah*	198	343
*pbv*		Reptilia	*Python bivittatus*	212	307
*xtr*		Amphibia	*Xenopus tropicalis*	196	182
*dvi*	Arthropoda	Insecta	*Drosophila virilis*	181	330
*dsi*		Insecta	*Drosophila simulans*	149	213
*sfr*		Insecta	*Spodoptera frugiperda*	122	221
*aae*		Insecta	*Aedes aegypti*	156	165
*dse*		Insecta	*Drosophila sechellia*	103	120
*der*		Insecta	*Drosophila erecta*	101	120
*dya*		Insecta	*Drosophila yakuba*	93	103
*hme*		Insecta	*Heliconius melpomene*	92	97
*pte*		Arachnida	*Parasteatoda tepidariorum*	148	257
*tur*		Arachnida	*Tetranychus urticae*	52	92
*rmi*		Arachnida	*Rhipicephalus microplus*	24	24
*hpo*	Nematoda	Secernentea	*Heligmosomoides polygyrus*	246	486
*cel*		Secernentea	*Caenorhabditis elegans*	261	439
*cbr*		Secernentea	*Caenorhabditis briggsae*	178	164
*str*		Secernentea	*Strongyloides ratti*	106	208
*sme*	Platyhelmintes	Rhabditophora	*Schmidtea mediterranea*	148	257
*egr*		Cestoda	*Echinococcus granulosus*	111	218
*sja*		Trematoda	*Schistosoma japonicum*	56	79
*emu*		Cestoda	*Echinococcus multilocularis*	36	68
*nve*	Cnidaria	Anthozoa	*Nematostella vectensis*	141	142
*hma*		Hydrozoa	*Hydra magnipapillata*	17	20

**Table 4:** Hairpin precursor and mature miRNA number in different virus family

ID	Group	Family	Name	Hairpin precursor	Mature miRNA
*bfv*	Group VI (ssRNA)	Retroviridae	Bovine foamy virus	2	4
*blv*	Group VI (ssRNA)	Retroviridae	Bovine leukemia virus	5	10
*ebv*	Group I (dsDNA)	Herpesviridae	Epstein barr virus	25	44
*hcmv*	Group I (dsDNA)	Herpesviridae	Human cytomegalovirus	15	26
*hiv*	Group VI (ssRNA)	Retroviridae	Human immunodeficiency virus 1	3	4
*hsv*	Group I (dsDNA)	Herpesviridae	Herpes simplex virus	37	51
*kshv*	Group I (dsDNA)	Herpesviridae	Kaposi sarcoma associated herpesvirus	13	25
*mcmv*	Group I (dsDNA)	Herpesviridae	Mouse cytomegalovirus	18	29
*mdv*	Group I (dsDNA)	Herpesviridae	Mareks disease virus type 1	32	62
*mghv*	Group I (dsDNA)	Herpesviridae	Mouse gammaherpes virus	15	28
*prv*	Group I (dsDNA)	Herpesviridae	Pseudorabies virus	14	18

Table 3 reports data related to different phyla of animal kingdom. Chordata possess the highest number of miRNAs in animal kingdom as shown in Fig. 2b, while Mammalia possess the highest number of miRNAs among Chordata (Fig. 2c). This is in agreement with the observation that Mammalia are the most complex phylum from evolutionary and body complex point of view. Among Insects, *D. melanogaster* possesses the highest number of miRNAs (362), while miRNA number average in Nematodes (522) results almost double than the one in Platyhelminthes (243)*.* In Cnidaria the existence of miRNA system silencing genes through DCR and AGO activation is well demonstrated. Cnidaria miRNAs act on the basis of a perfect miRNA/mRNA complementarity, as reported for plants [60]. This mechanism was already present in UrEumetazoa, the common ancestor of Cnidaria and Metazoa, [61]. The evidence that miRNA machinery has been maintained during evolution in different divisions and phyla supports the idea that miRNAs have been a fundamental key in evolutionary steps [62].

miRNA database also reports miRNA number in subcellular organisms including viruses. Table 4 shows miRNA number in ssRNA and dsDNA virus groups. miRNA number average in dsDNA group (56) is higher than the one in ssRNA group (9).

From the analysis of miRNA number in various organisms we can conclude that miRNA number identified and catalogued for each animal group decreased from complex multicellular organisms (Chordata) to less complex organisms such as Cnidaria, according with studies reporting a relationship between the increase in number of miRNAs and body evolution of organisms [63]. As expected, viruses showed the lowest miRNA number (Fig. 2c).

Knowledge on the origin and divergence of miRNAs paves the way for a better understanding of the complexity of the regulatory network that they participate in.

## miRNA flow from plants to animals

miRNAs are widespread and highly conserved in plant species. Initially, they have been identified in arabidopsis*,* rice, tobacco and maize suggesting that miRNAs may have the same ancestor in very early evolution. However, the expression of different miRNAs and their copy number in plant genome may differ, and therefore there is a very high rate of divergence occurring for some miRNAs, even if some are highly conserved. As reported above, miRNAs derive from dsRNA precursors processed by DCR. If the processing is followed by methylation, as in plant miRNA biogenesis, an inheritable epigenetic modification occurs. Indeed, plant methylated miRNAs are resistant to periodate, while animal miRNAs are sensitive to periodate [35]. Then, exploiting this peculiarity it is possible to distinguish between plant and animal miRNAs. Several studies have been carried out in animals undergoing different diets with the aim of clarifying the intriguing question whether plant miRNAs can enter into animal cells, exerting physiological functions. In particular, the characterization of small RNAs in healthy Chinese donors showed that plant miRNAs represented about 5% of mammalian miRNAs; miRNAs cloned from donor serum were resistant to periodate, suggesting they were plant miRNAs, probably coming from food intake. As a confirmation of this hypothesis, the concentration of plant miRNAs was found to be higher in the serum of rice-fed mice compared with chow diet-fed mice [64]. However, despite variations of diet components, this percentage was never above 10%; moreover, no differences in miRNA content were observed comparing diets composed of raw or cooked food, indicating that miRNAs are heat resistant. It is also suggested by different authors that plant derived miRNAs can survive the animal digestion system, absorbed and transferred into blood, circulate through animal body, regulating animal gene expression as endogenous miRNAs [65–67].

Zhang group firstly reported that food-derived miRNAs are accumulated in human plasma micro-vesicles (exosomes) that can transport them throughout the body, thus being widespread in various organs and tissues with the consequent inhibition of specific gene expression. In particular, rice miR168a has been suggested to target the mRNA of the low-density lipoprotein receptor adaptor protein 1, and rice-fed mice showed a reduction in receptor expression in blood and liver. Evidences also indicated that the integration of exogenous miR168 from plants can contribute to modulate dyslipidemia in mammals [64, 68]. The presence of rice miRNAs in human plasma was further supported by Wang, who using next generation small RNA sequencing datasets of human serum samples, identified several exogenous small RNA species from gut microbiota and plant species [69]. Hence, the plant specific miR172 has been found in the stomach, intestine and serum of mouse fed with RNA extracted from brassicaceae, suggesting that plant miRNA can survive in the circulation and gastrointestinal tract in mice [65]. An in silico study, aimed at evaluating the presence of plant food derived miRNAs in mammalian breast milk, showed that 35 plant miRNAs were found in human exosome samples [70]. Analysing breast milk from healthy donors by PCR, several plant miRNAs were found in human milk, hypothesising that these miRNAs may potentially influence the biological pathway in infants [71]. Several other studies confirmed the finding of plant miRNAs in tissue of different species such as pig [67], panda [72] and silkworm [73]. Plant miRNA relevance in the prevention or treatment of human diseases, including cardiovascular diseases [74], tumors [75, 76], chronic-inflammation [77], influenza [78], and pulmonary fibrosis [79] was proposed. In particular, a study by Zhou, provided evidence that the highly stable plant miR2911 could be taken up via gastrointestinal tract and directly target multiple viral genes of various influenza A viruses, and thus counteract viral infections [78]. miR2911 is an atypical miRNA deriving from 26S ribosomal RNA encoded by honeysuckle (Lonicera japonica), a traditional Chinese herb widely used to effectively treat influenza and other pathogen infections. Honeysuckle decoction has been shown to suppress the replication of influenza virus, to exert an anti-viral effect against influenza in human body through the uptake of miR2911 [80]. Employing the mouse model, the delivery and accumulation of miR2911 in various organs has also been demonstrated. Moreover, higher levels of miR168 was found in mice fed with miR168 combined with honeysuckle diet than in mice feed with miR168 alone, suggesting that dietary habit may influence the absorption of miRNAs [81]. In the recent years, several studies revealed that human, bovine, pig and rat milk, as well as other biological fluids, contained miRNAs [82, 83]. In particular, the miRNA microarray analyses of bovine milk revealed the presence of 79 and 91 miRNAs in the exosomes milk and in the supernatant whey derivatives, respectively [84]. Exosomes are naturally 30–100 nm nanovescicles containing different biomolecules including nucleic acids such as miRNAs and other ncRNAs, secreted into the extracellular liquids by different types of cells [85]. Exosomes play an important role in cellular trafficking and intercellular communication in multicellular organisms; these physiological stable nanovescicles might exert their trans-species modulation by acting as cargo for various RNA types, despite RNAase activity [86]. The cell-to-cell communication mediated by exosomes transferring genetic information was first addressed by Valadi, and later confirmed by other authors reporting that commercial milk contained stable exosomes that remained intact in the gastrointestinal tract and exerted an immunoregulatory effect [87, 88]. Exosomes from human or animal cells can be harvested from cell culture liquid, blood, urine, and other body fluids. Analogous fluids in plants are not so easily collected. However, some authors suggested that exosomes may have originally evolved in plants as a mean of cell–cell communication between plants, regulating innate immune defences in response to pathogen invasion [89]. The same authors also speculate that exosomes liberated from digested edible plants may be a way of cross-species communication. Recently, plant derived exosome-like nanovescicles, having a structure similar to those of mammalian exosomes, have been characterized in different edible plant [90]. These nanovescicles can be absorbed in the mammalian gastrointestinal tract and have the potential to mediate plant–animal intercellular communication [91, 92]. For example, nanoparticles from four edible plants (grape, grapefruit, ginger, and carrot) have been shown to possess anti-inflammatory properties. One recent study demonstrated that plant derived exosome-like particles are taken up by the gut microbiota of mice, protecting them from symptoms of colitis [93].

However, the hypothesis that diet derived exogenous miRNAs can exert any influence on human gene regulation has yielded a lot of controversies. Indeed, several sources of DNA contamination, artefacts and false positive results have been suggested by different authors [94, 95]. In particular, little or low measurable uptake of plant miRNAs by PCR in human and primates after feeding studies have been found [96, 97]. Moreover, Dickinson attempted to validate Zhang results, but found little evidence of dietary uptake of miR168 after rice feeding [98]. The different points of view on the possible cross-kingdom exchange between plants and animals are discussed in a recent publication where Zhang and Witwer share their realities of dietary miRNAs by answering five questions related to this controversial field [99].

### The influence of miRNAs contained in food on development: the case of honeybee

**Fig. 3:**
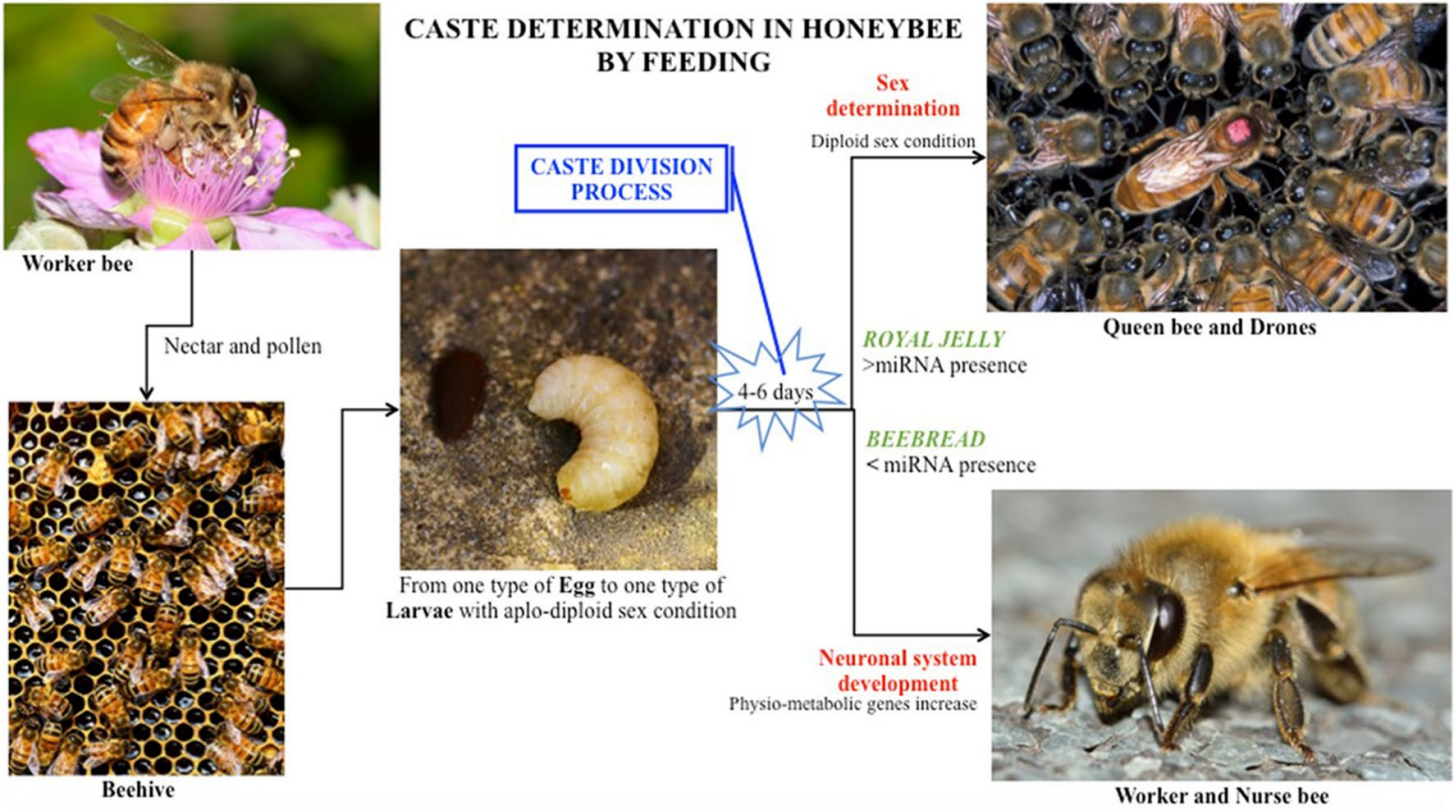
*Apis mellifera* life cycle and the influence of food on caste development

In relation to the acquirement of plant miRNAs through food intake, we report the example of honeybee (*Apis mellifera*). Honeybee is a paradigm of miRNA-related epigenetic control of phenotype expression. Honeybee is an eusocial insect living in a social system rigorously divided into castes, where each bee has its own well-defined role. The caste division and fate determination are important for understanding the epigenetic control in organism development. How castes evolve has at present not fully answered. At this regard, the relationship between miRNA and fate determination of larvae has been highlighted. All the bees develop from one type of egg, thus having the same genome, female being diployd and male being aployd. Female honeybees are divided further into two castes, queens and workers, which differ in morphology, physiology and social function. Larvae receive a series of specific food stimuli including the administration of different miRNA sets, leading to morphological and structural changes [100]. In particular, during the 4th to 6th day of larval development honeybees are fed with two types of mush: (a) royal jelly, a glandular secretion of nurse bees, causing the stimulation of differentiation to queen bee and drones, the latter differing from queen because of their aployd genome; (b) beebread, composed of a fermented mixture of plant nectar and pollen, leading to the worker phenotype [101, 102]**.** Several components of the larval diet have been suggested to influence caste development, even if the influence of food on the developmental fate of honeybees is not fully understood. Recently, evidences have been provided for a previously uncharacterized regulatory mechanism of worker bee development, which can be partially attributed to the plant miRNAs contained in beebread feeding young larvae. At the initial stage, the larvae fed with the same food expressed basal miRNAs that are mainly aimed at the control of neuronal non-physio-metabolic genes. At the 4–6th day of larval development the administration of royal jelly is related to the expression of physio-metabolic genes in queen bee [103] (Fig. 3). It is worth to underline that the amount of miRNAs in beebread is 7–215 fold higher than in royal jelly, and that miRNAs are prevalently of plant origin. Studies aimed at characterizing miRNA expression in bees, indicated that miR162, a typical plant miRNA, is highly express in worker bees [102]. Beebread alimentary choice involves greater gene expression enrichment as compared to royal jelly, whose consequence is the development of the neuronal system in the worker bees at the expenses of the physio-metabolic pathways [104]. This is in agreement with the fact that miRNAs are differentially expressed during the caste development of bees. For example, among workers miR210, associated with memory and learning, is higher expressed than in queen bee [105]. Moreover, queen larvae expressed ame-let-7 twice than worker bee larvae, whereas the expression of ame-miR-263 in queen larvae was only one fifth of that in worker bee larvae [101]. These data are supported by studies showing that miRNAs contained in beebread used for feeding the larvae of prospective queens were capable of direct adult differentiation towards worker bees. The addition of miR184, abundant in beebread, to food of queen larvae influenced the morphology of adult bees in the direction of the worker phenotype.

## miRNAs in clinical trials

miRNAs are small molecules that have the ability to regulate the expression of several mRNAs, and their aberrant expression has been linked to the development of multiple diseases. One only miRNA has the potential capability to regulate biological pathways that are disrupted in patients, indeed they act as intracellular mediators and can potentially modify the expression of thousands genes. This is why miRNAs are considered one of the most promising therapeutic approach to be developed for the future clinical researches [106, 107]. Indeed, their use as drugs could be particular advantageous for the therapy of diseases that are provoked by multifactorial events, such as cancer. In patients the level of downregulated tumour suppressed miRNAs could be normalized by their re-expression using synthetic miRNAs, while the upregulated oncogenic miRNAs could be silenced by antisense mediated inhibition [108, 109]. Several miRNAs have been proposed as therapeutics. miRNA targeted therapeutics that have reached clinical development, and related diseases for which they can be used are reported in Table 5 [110, 111].

**Table 5:** miRNAs in clinical trials

miRNA	Drug name	Disease
miR-34	MRX34	Multiple cancers
miR-92	MRG110 MGN-6114	Heart failure/wound healing
miR-16	mesomiR-1	Mesothelioma, lung cancer
miR-122	Miravirsen RG101	Hepatitis C virus
miR-29	MRG201 MGN4220	Keloid, fibrous scar tissue formation
miR-21	RG012	Alport syndrome
miR-155	Cobomarsen (MRG-106)	T-cell lymphoma/mycosis fungoides
miR-143/145	MGN2677	Vascular disease
miR-451	MGN-4893	Polycythemia vera
miR-378	MGN5804	Cardiometabolic disease
miR-15/miR-195	MGN-1374	Post-myocardial infarction remodelling
miR-208	MGN9103	Heart failure

## Conclusions

miRNAs are the smallest nucleotide molecules able to regulate gene expression at post transcriptional level found in both animals and plants thus their origin, by the evolutionary point of view, is by far much older than the appearance of human on Earth. Indeed, they were conserved in different species, because they are involved in fundamental processes for life, growth and development of living organisms. Plants have developed mechanisms of growth and defense much more efficient than animals because they need to respond more quickly and effectively to environmental stress and external attacks, lacking an efficient immunosystem. This could explain why plant miRNAs are often more effective in their biological functions than the animal ones. miRNAs are generally transmitted to the progeny in a quite stable manner. However, further to the vertical transmission, miRNAs can undergo horizontal transmission among different species, in particular between plant and animals through food. Furthermore, the transmission of miRNAs to plant feeding animals such as bees makes the study of plant miRNAs fundamental to shed light on the biological role of these molecules across kingdoms.

In the last years, an increasing number of both in vitro and in vivo studies reported that miRNA passage can occur through feeding and that in mammals, plant miRNAs survive the gastro intestinal digestion, absorbed, and transferred by blood into host cells where they exert they functions modulating gene expression. Adaptation of miRNAs into the new organisms, where exogenous miRNAs have been maintained and integrated due to the positive changes on the metabolism, has been suggested.

The majority of human medicines and supplements derive from plants and a regular consumption of plant food is suggested for their beneficial effects in the prevention of metabolic diseases, cancers and age related functional disorders. So far, these beneficial effects have been generally attributed to the content of secondary metabolites, whereas mechanisms regarding other components remain unclear. Therefore, in light of the above reported studies miRNA could result another component for the medical properties of plants. However, the question on how plant miRNAs can be transferred to animals, including humans, and how they are able to effectively regulate gene expression in a cross-kingdom manner is still under debate.

sncRNAs have been mainly studied in mammals characterizing their sequences and molecular targets as available in public databases. The herein presented studies provide evidence that miRNA situation is much more complex than the static situation reported in databases. Indeed, miRNAs may have redundant activities, variable sequences, different methods of biogenesis, and may be differently influenced by external and environmental factors. In-depth knowledge of mechanisms of synthesis, regulation and transfer of plant sncRNAs, such as miRNAs and siRNAs, to other species can open new frontiers in the treatment and therapy of many human diseases, including cancer.

## Data Availability

Not applicable.

## Abbreviations

AGO: argonaute
DCR: dicer
DCL: dicer-like
miRNAs: microRNAs
pre-miRNAs: miRNA precursors
ncRNAs: non-coding RNAs
pri-miRNAs: primary miRNAs
RISC: RNA-induced silencing complex
sncRNAs: small ncRNAs
siRNAs: short interfering RNAs
